# Influence of Friction Stir Surface Processing on the Corrosion Resistance of Al 6061

**DOI:** 10.3390/ma15228124

**Published:** 2022-11-16

**Authors:** Ibrahim H. Zainelabdeen, Fadi A. Al-Badour, Rami K. Suleiman, Akeem Yusuf Adesina, Necar Merah, Fadi A. Ghaith

**Affiliations:** 1 Mechanical Engineering Department, King Fahd University of Petroleum and Minerals, Dhahran 31261, Saudi Arabia; 2 Interdisciplinary Research Center for Advanced Materials, King Fahd University of Petroleum and Minerals, Dhahran 31261, Saudi Arabia; 3 School of Engineering and Physical Sciences, Heriot-Watt University, Dubai 38103, United Arab Emirates

**Keywords:** friction stir processing, pinless tool, surface processing, corrosion resistance, saline environment, Al 6061

## Abstract

In this work, friction stir processing using a pinless tool with a featured shoulder was performed to alter the surface properties of Al 6061-O, focusing on the effect of tool traverse speed on surface properties, i.e., microstructure, hardness, and corrosion resistance. All processed samples showed refinement in grain size, microhardness, and corrosion resistance compared to the base material. Increasing tool-traverse speed marginally refined the microstructure, but produced a significant reduction in microhardness. Electrochemical impedance spectroscopy, linear polarization resistance, and potentiodynamic polarization were used to evaluate the effect of the processing conditions on corrosion behavior in a saline environment. All corrosion test results are found to agree and were supported with pictures of corroded samples captured using a field emission scanning electron microscope. A remarkable reduction in the corrosion rate was obtained with increasing traverse speed. At the highest traverse speed, the corrosion current density dropped by approximately 600 times when compared with that of the base alloy according to potentiodynamic polarization results. This is mainly due to the grain refinement produced by the friction stir process.

## 1. Introduction

Aluminum-based alloys are utilized in a variety of advanced commercial applications such as automotive, defense, marine, and aerospace. This is ascribed to their outstanding characteristics, such as a high strength-to-weight ratio, high ductility, and good corrosion resistance. In addition, the resistivity of aluminum alloys against corrosion is mainly attributed to the oxide layer formed when the alloy is exposed to the environment. It is, however, believed that aluminum-based alloys exhibit low corrosion resistance when exposed to saline media [1]. Along with bad corrosion resistance in saline media, aluminum-based alloys suffer from bad surface characteristics such as wear resistance and low hardness. Therefore, the adoption of aluminum alloys in saline environments is restricted [2]. The impact of grain size on the mechanical and physical characteristics of different alloys is well documented and it is known to be a vital determinant of many microstructure-dependent properties [3]. Therefore, grain size refinement is among the most adopted approaches for improved material properties. Thus, several alternatives have been developed over the last few decades to produce refined grains in a feasible way such as severe plastic deformation techniques [4]. One such technique is friction stir processing (FSP), which has drawn more attention because of its many advantages [5]. Most severe plastic deformation techniques result in bulk material processing or the altering of the initial part shape, Moreover, a special surface pre-processing/treatment is required [6]. FSP was developed on the same working principles as friction stir welding (FSW), where a rotating tool, consisting of two parts—a shoulder with a larger diameter and a pin—is pressed against neighboring ends of sheets or plates that need to be linked and then moved along the line of the joint [7]. FSP uses the same principle in processing the surface and subsurface of a single bulk workpiece, thereby modifying the surface properties by the dynamic recrystallization process encountered in FSW. The microstructural evolution and mechanical properties after FSP of various alloys have been extensively studied over the last two decades. Nevertheless, the effect on corrosion resistance of FSP has not received the same attention. Several studies have indicated that the corrosion susceptibility may either diminish or increase for different aluminum alloys after severe plastic deformation, as reported by Mehdi et al. [8]. It was suggested that the corrosion behavior primarily depends on the severe plastic deformation processes, working material alloying elements, and process conditions. For example, Surekha et al. [9,10], in two separate studies, have investigated the influence of traverse speed, rotation speed, and the number of passes on the corrosion resistance of FS-processed 2219 AA. Their results showed that the corrosion susceptibility was influenced by changing the number of passes and rotating speeds, rather than the traverse speed. They reported an enhancement in corrosion resistance with the increase in tool rotational speed and number of passes which was ascribed to the reduction in the intermetallic phases. Conversely, Eldeeb et al. [11] reported that the traverse speed has more influence on the corrosion resistance of the stirred zone in comparison to the tool rotational speed of friction-stir-processed Al6061-O. Esmaily et al. [12] have reported enhancement in the corrosion behavior of 6005 AA after deploying multi-pass FSP. Reddy et al. [13] also compared the corrosion behavior and wear resistance of FS-processed A356 with that of cast material. It was revealed that FSP resulted in outstanding corrosion and wear resistance. They explained the improvement in the wear resistance by the homogeneous dispersion of silicon particles through the aluminum matrix, whereas the reduction in corrosion rate was attributed to the formation of an intact passive film.

It should be noted that most of the literature on FSP focuses on studying the impact of changing parameters on the microstructural features, mechanical, and corrosion characteristics of full penetration FSP. However, to the authors’ knowledge, few investigations have been carried out on the effect of friction stir surface processing parameters using a pinless tool on the microstructure, and consequently, the corrosion resistance, associated with the newly developed surface, with a focus on aluminum and its alloys. To rectify this, the impact of varying the tool traverse speed during FSP on the grain structure, microhardness, and corrosion behavior of 6061-O AA base metal is studied herein in detail.

## 2. Materials and Methods

**Figure 1:**
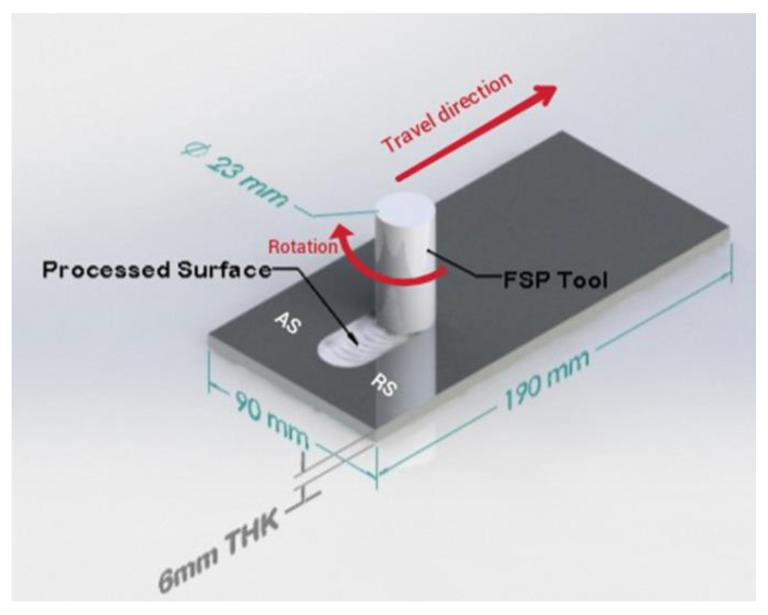
The schematic diagram for friction stir processing.

The workpieces utilized in the current study are an aluminum alloy 6061-O grade with dimensions of 190 × 90 × 6 mm^3^ (Figure 1). The chemical composition of the workpieces was obtained using spark spectrometry and is shown in Table 1. A novel pinless FSP tool was designed and fabricated for this study from 4140 alloyed steel. The tool was heat-treated to a hardness ranging between 52 and 54 HRC. The FSP tool has a shoulder diameter of 23 mm with two circular groves, which were machined such that they will enhance the material flow under the tool shoulder and increase the contact area. Figure 1 is a schematic demonstration of the FSP process, showing the pinless rotating tool traversing the workpiece to be processed. A literature search showed that a rotation speed of 1000 rpm yields better properties for different grades of aluminum alloys [14,15,16,17,18]. Therefore, the rotation speed was fixed at 1000 rpm, while the traverse speed was varied from 100 to 250 mm/min. To improve the forging action, the tool was tilted by 3° towards the trailing side [19], with a plunge depth of 0.2 mm to assure sufficient forging force. FSP was carried out using a research-based three-axis RM-1 friction stir welder (MTI, West Washington, South Bend, IN, USA) under position control mode. Accordingly, process conditions and sample designations are shown in Table 2. After FSP, the base metal and processed samples were sectioned from the processed zone into a suitable size for subsequent characterization and corrosion testing; thereafter prepared by grinding using silicon carbide papers with grit sizes varying from 240 to 1200. Consequently, a polishing process using alumina and diamond paste was performed to obtain a mirror-like finish followed by etching using 2 g of sodium hydroxide in 100 mL of distilled water to reveal the grain structure.

### 2.1. Characterization and Mechanical Properties

An optical digital microscope (Olympus, DSX 510, Tokyo, Japan) was utilized to capture images of the etched surfaces, while Olympus stream software was used to determine the grain size of the friction-stir-processed surface as well as the base material, following ASTM 112-13 [20]. A line intercept procedure (average of horizontal and vertical intercept lengths) was adopted in measuring the average grain size as per the recommendation of the ASTM-112-13 standard. More insights into grain morphology parameters, as well as the method used to measure grain size, are shown in Figures and Tables S1–S4 (Supplementary Material). In addition, images of corroded samples were acquired using a field emission Scanning Electron Microscope (FESEM) (Quanta 250, Bruno, Czech Republic). Analysis of the present phases for the base and all processed samples was performed using X-ray Diffraction (XRD) in a Bruker diffractometer (D2 PHASER) that operated at 30 kV with CuK_α_ (λ_α_ = 1.54 Å). A micro indenter (Micro Combi, CSM Instruments, Peseux, Switzerland) was utilized to measure the hardness and elastic modulus of the base and processed samples. A pyramidal indenter was used at a normal force of 3 N for 10 s dwell time. The test was repeated six times and the average values of microhardness and elastic modulus were recorded.

### 2.2. Electrochemical Analysis

All electrochemical measurements, consisting of electrochemical impedance spectroscopy (EIS), linear polarization (LPR), and potentiodynamic polarization (PDP) measurements, on the base sample as well as the processed samples were carried out on the traditional three-electrode configuration which includes a graphite counter electrode and a saturated calomel electrode (SCE) reference electrode. A Gamry Instruments Reference 3000 potentiostat/galvanostat was employed for data collection, while Echem Analyst 6.0 software was used for electrochemical data analysis and fitting. All working electrode samples i.e., the processed samples, were machined from the middle of the processed zone into a suitable size and ground using silicon carbide papers up to 800 grit and thereafter masked such that only 1 cm^2^ of the processed zone was exposed to the 3.5 wt.% NaCl at 23 °C for 15 days. Before conducting the corrosion measurements, all samples were cleaned using distilled water and acetone. EIS tests were conducted using a frequency range of 10^−2^ to 10^5^ Hz and an AC voltage of 10 mV. PDP tests were carried out at a scanning rate of 0.125 mV/s using an applied potential varying between –0.20 V, cathodically, and 0.50 V, anodically, under open circuit potential (OCP). Thereafter, Tafel parameters were obtained from PDP curves. LPR measurements were acquired at a scanning rate of 100 mV/s between +25 and –25 mV under OCP.

## 3. Results and Discussion

### 3.1. Effect of Traverse Speed on Forging Force and Torque

**Figure 2:**
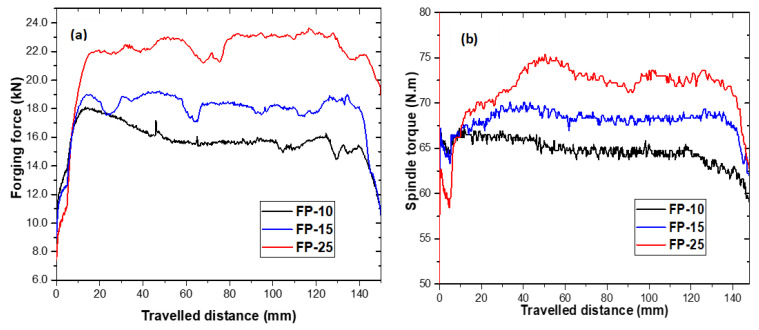
Effect of traverse speed on (**a**) forging forces and (**b**) spindle torque.

The effect of increasing traverse speed from 100 to 250 mm/min on forging force is presented in Figure 2a. It is obvious from these results that the forging force increases with increasing traverse speed, reaching 22 kN at 250 mm/min. The increase in forging force with traverse speed is attributed to the heat input reduction, which makes the area around the tool harder and thereby raises the material flow stress and consequently the forging forces [21,22,23] for maintaining the tool plunging depth. Figure 2b shows that the traverse speed has an even higher impact on the generated torque. The considerable increase in the measured torque with the rise in traverse speed is also attributable to higher FSP-induced flow stresses [24]. It is evident that increasing the tool traverse speed under constant rotation speed results in a heat-input reduction, whereas increasing the tool rotation speed and fixing the tool traverse speed results in increasing the heat input during the friction stir processing, and vice versa. Accordingly, the heat input for each process parameter is obtained from the measured spindle torque, tool rotation, and traverse speeds (Equation (1)) [25].

(1) $$H\;=\frac{P}{v}=\frac{T\;\omega }{v}$$

where H is the heat input in J/mm, T is the average measured spindle torque in N·m, ω is the tool rotation speed in rad/s, and v is the tool traverse speed in mm/s.

It is worth noting that the heat input equation (Equation (1)) neglects the efficiency of the heat transfer process. Moreover, it assumes that the frictional work of the tool is completely converted into heat, resulting in material softening.

### 3.2. Microstructural and Mechanical Properties Analysis

**Figure 3:**
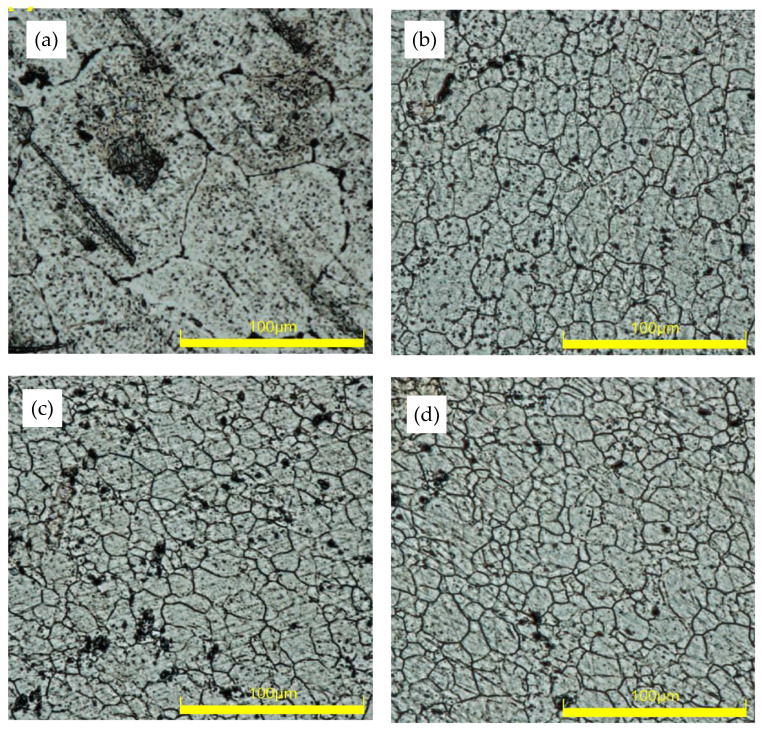
Optical micrograph of the samples showing grain morphology of (**a**) unprocessed base, (**b**) FP-10 (**c**) FP-15, and (**d**) FP-25.

The optical microscopic images of as-received (base), and FS-processed for the samples at various traverse speeds, 100, 150, and 250 mm/min, are presented in Figure 3a, Figure 3b, Figure 3c, and Figure 3d, respectively. It is worth noting that the only source of heat during this investigation was that of the tool shoulder interaction with the workpiece material. Compared to the base metal, samples processed at different traverse speeds exhibited substantial grain refinement. Adopting FSP at a traverse speed of 100 mm/min significantly reduces the average grain size of the base specimen from 93.93 µm to 17.74 µm. Additionally, the grain size exhibits a slight reduction with increasing traverse speed, from 16.38 µm for the sample processed at 150 mm/min to 15.58 µm for the sample processed at the highest speed of 250 mm/min. Grain refinement with increasing traverse speed during friction stir processing has been extensively reported in the literature and has been attributed to a heat-input reduction [26,27]. It is worth noting that microstructure evolution is sensitive to heat input, where higher heat input is not only accompanied by grain growth but also a significant change in grain morphology [28]. In addition, the relationship between the grain growth upon cooling and heat input induced by the severe plastic deformation during friction stir processing can be explained through the following equation (Equation (2)) [29].

D^2^ − D^2^_0_ = At exp (−Q/RT) (2)

where D and D_0_ are the initial and deformed grain size, respectively, A is a constant, T is the peak temperature, Q is the activation energy, and t is the time to cool down to 448 K [29]. Furthermore, it was reported that dynamic recrystallization is the main mechanism of grain refinement during FSP where the processed zone experienced high strain rates and high peak temperatures [30].

**Figure 4:**
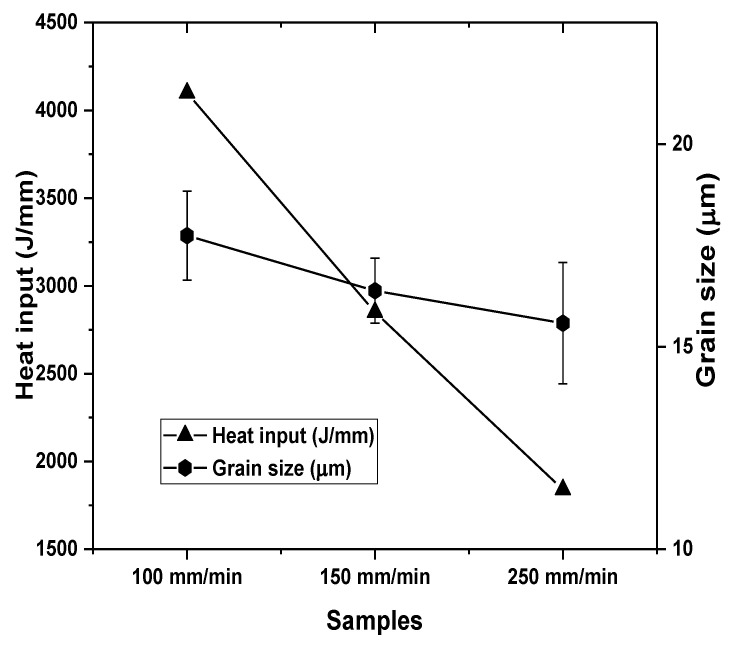
Effect of tool traverse speed on heat input and processed surface grain size.

Figure 4 presents the effect of tool traverse speed on calculated heat input using (Equation (1)) and the measured grain size.

**Figure 5:**
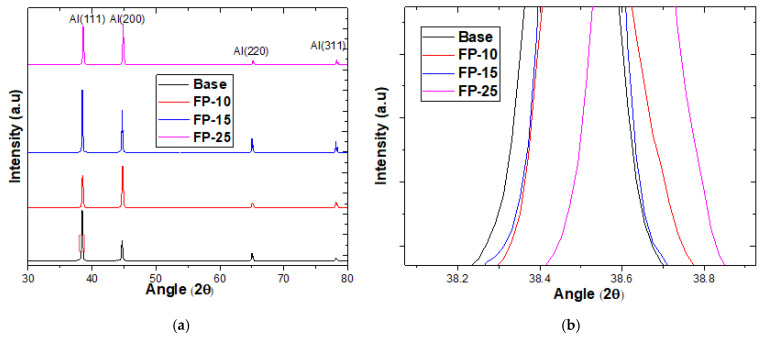
(**a**) XRD spectra for all samples before and after FSP at different traverse speeds. (**b**) zoom in at (111) peak.

Figure 5 shows the influence of varying FSP traverse speed on the XRD patterns. As Figure 5a shows, only the α-Al phase was detected for the base material and processed samples. Furthermore, little change in diffraction peaks was observed, which confirmed that no secondary intermetallic phases precipitated because of plastic deformation induced by FSP. Interestingly, the samples after processing showed an appreciable shift toward high diffraction angles, and the sample processed at the highest processing speed showed the most pronounced shift, as can clearly be seen in Figure 5b. To the best of the authors’ knowledge, a shift in aluminum peaks after FSP was not reported before; it has, however, been reported for magnesium and copper after FSP. This phenomenon of peak shifting was ascribed to the reduction in lattice parameters that causes compressive stresses [31,32].

**Figure 6:**
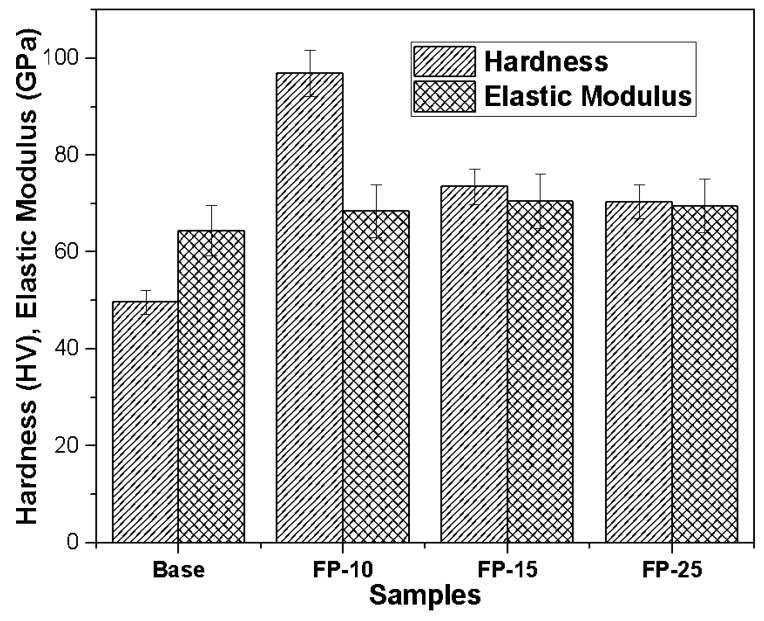
Impact of increasing tool traverse speed on microhardness and elastic modulus.

The microhardness and elastic modulus of the base sample was clearly much lower compared to that of the processed samples under all processing conditions, as shown in Figure 6. The microhardness of samples FP-10, FP-15, and FP-25 were improved by 96, 48, and 40%, respectively, compared to that of the base metal. The enhanced hardness is ascribed to the high heat input that may have resulted in the reduction of coarse secondary strengthening precipitate [33]. It should be mentioned that, though the grain sizes of the processed materials at different speeds are comparable (Figure 3), the measured hardnesses are different. Researchers have shown that, in precipitation-hardened aluminum alloys, grain refinement is not the dominant strengthening mechanism [34]. Other researchers have found that the effect of grain size of precipitated aluminum alloys on hardness is insignificant [35]. In addition, the elastic modulus of the base sample was around 64 GPa while, after performing FSP at the lowest speed, the elastic modulus increased to around 68 GPa. Moreover, the elastic modulus for the samples processed at moderate and high speeds showed only a small further improvement to approximately 70 GPa. The improvement in the elastic modulus may be attributed to dynamic recrystallization [36], which leads to reduced dislocation mobility, therefore, minimizing elastic strain.

### 3.3. Electrochemical Behavior

#### 3.3.1. Electrochemical Impedance Spectroscopy

Electrochemical impedance spectroscopy (EIS) has been extensively used in the investigation of the metal-electrolyte interface, surface responses, oxides films formation, passivation, corrosion kinetics and mechanism, and coating protective effectiveness to metallic substrates [37,38]. Thus, in order to investigate the effects of FSP parameters on the kinetics and characteristics of the electrochemical process, EIS measurements were carried out on the base sample and all processed samples.

**Figure 7:**
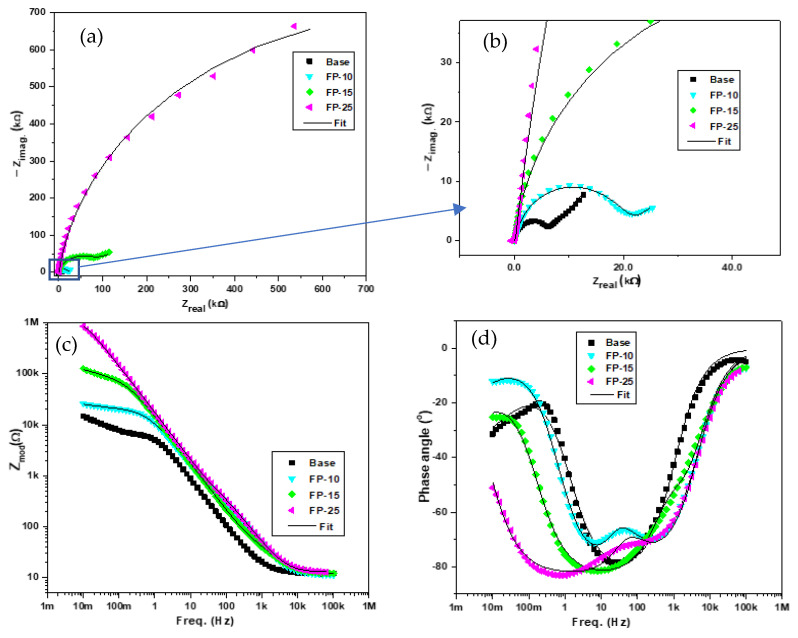
Plots of the EIS experimental and fitted data for all samples showing the (**a**,**b**) Nyquist, (**c**) bode, and (**d**) phase angle plots.

Figure 7a–d displays the Nyquist curves, phase angle, and modulus of the base sample and all processed samples. The Nyquist plots depicted in Figure 7a,b show a relatively large incomplete semicircle at the high-frequency region, while the low-frequency region demonstrates a diffusion tail. The larger incomplete semicircle at the high-frequency region in the Nyquist plot of processed samples may be ascribed to a charge transfer reaction at the boundary between the thin film formed on the aluminum surface and the NaCl solution [39]. However, the occurrence of the small tail at the low-frequency region of the spectra is attributed to diffusion-controlled processes [40]. The depression phenomenon under the x-axis can be clearly identified through the incomplete semicircle behavior of the arcs, which is probably caused by surface heterogeneities resulting from possible corrosion products and frequency dispersion [41]. As can be observed in Figure 7a, the arc diameter rises with increasing traverse speed. In addition, the smallest diameter was observed for the base sample, while the sample processed with the highest traverse speed (FP-25) demonstrates the highest semicircle diameter. Moreover, the variation in the semicircle diameter between the base sample and the sample processed at the lowest traverse speed (FP-10) was remarkably low compared to the deviation in semicircle diameter between the sample processed at moderate velocity (FP-15) and that fabricated at the highest velocity (FP-25). Interestingly, the variance in semicircle diameter between the base sample and (FP-25) sample was not even distinguishable without zooming the base sample area in the Nyquist plot, as seen in Figure 7b. A huge increment in the semicircle diameters revealed the significant impact of process conditions on corrosion behavior. Furthermore, the increase in the radius of the semicircle with the processing can be interpreted by an enhancement in the surface protection due to the rising stability and compactness of the passive film formed [42,43]. Therefore, the enhancement in the corrosion resistance was found to follow the rank of FP-10 < FP-15 < FP-25, and demonstrated a markedly improved resistance compared to that of the unprocessed sample.

The bode plots for all samples are presented in Figure 7c. It is evident from the figure that the total impedance for all the processed samples is remarkably higher than that of the base sample, which reflects the increased modulus impedance of the processed samples. In addition, the total impedance revealed a significant increment by increasing the traverse speed. Moreover, the deviation in the total impedance was at its lowest when comparing the base material with the sample processed at the lowest traverse speed; however, with increasing traverse speed, the deviation between samples processed at the intermediate and lowest speed was much higher. Furthermore, a huge variation was observed between the intermediate-speed sample (FP-15) and the sample processed at the highest speed (FP-25), which indicates a superior corrosion resistance for the sample processed at 250 mm/min compared to all other samples.

Figure 7d displays the typical Phase angle-frequency plots for both the processed and base samples. The frequency of the phase angle maxima of the base sample is 25 Hz at −78°. In contrast, the (FP-10) sample demonstrated two time constants, the first shifted toward a frequency of 6.4 Hz at approximately −71° and the second shifted toward a frequency of 241 Hz at −69°. Additionally, samples (FP-15) and (FP-25) exhibited phase angle maxima that are shifted toward frequencies of 8 and 0.78 Hz at −81 and −83°, respectively. It is worth noting that observing the phase angle maxima for the base sample at higher frequency suggests weakness in the protective barrier [44]. Moreover, it can be noticed that the base sample demonstrated a single time constant and upon increasing the traverse speed two time-constant characteristics can be observed. Similar behavior was also observed in [45]. Furthermore, more peak broadening indicates an enhancement in the passive protective barrier over a wide frequency range and a consequent corrosion resistance improvement [44,46].

**Figure 8:**
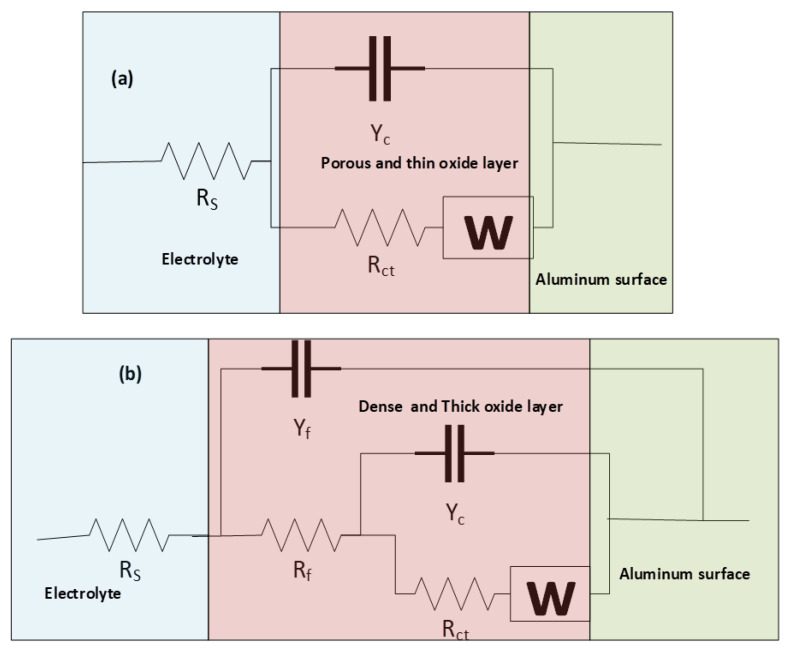
Proposed equivalent circuits of (**a**) base, and (**b**) processed samples.

All extracted impedance curves were fitted to appropriate corresponding circuits for further numerical estimation of the barrier characteristics of processed surfaces and properties of active corrosion protection. Accordingly, two equivalent circuits were proposed; the first one (Circuit a) was used to stimulate the corrosion behavior at the base surface as shown in Figure 8a. This circuit included a charge transfer resistance (R_ct_) that serially connected to a Warburg element (W) and was in parallel to a constant phase element for double-layer capacitance (CPE_dl_), where both (W, R_ct_//CPE_dl_) components were connected in series with the solution resistance (R_S_) component between the reference and working electrodes. The second circuit illustrated in Figure 8b (Circuit b) was used to simulate all the electrochemical processes at the surface of processed samples and contained an R_ct_ that linked in series to a Warburg element (W) and both components were in parallel with a constant phase element accounting for the double-layer capacitance of the inner barrier layer (CPE_dl_), and the aforementioned three components were connected in series with a film resistance (R_f_) component. Further, W, R_ct,_ CPE_dl,_ and R_f_ were connected in parallel with the constant phase element of the outer passive film (CPE_f_), and the previous circuit was connected in series with the solution resistance between the reference and working electrodes (R_S_).

The charge transfer resistance (R_ct_) depicted in Table 3 exhibited a rapid increment for all processed samples over the base material. In addition, it is evident from the fitted results that the charge transfer resistance increases with increasing traverse speed, which can be ascribed to the passive film formed at the metal/electrolyte interface [47]. The presence of this film isolated the aluminum metal surface from the corrosive ions attacks and thereby obstructing any further transfer of charge or mass. It should be noted that there is a remarkable difference between the charge transfer resistance (R_ct_) and film resistance (R_f_) particularly if the processing was carried out at high speeds and this may be attributed to the key role of the formed oxide film on the metal surface in mitigating the corrosion process at a particular speed [48]. Moreover, the huge variation between the outer and inner resistances indicates that the resistance provided by the inner barrier is much higher when compared with the outer film [49]. Generally, the Warburg element is added to simulate the diffusion effect as indicated by the straight line of a slope close to 45° to the impedance real axis. This can be observed with the unprocessed sample and gradually reduces for samples processed at 100 and 150 mm/min as seen in Figure 7a,b. It is expected that a further decrease will be experienced at the highest traverse speed sample i.e., FP-25 if the test was allowed for a much lower frequency. Considering the Warburg impedance, it is obvious from the table that the lowest Warburg impedance was obtained at the highest traverse speed of 250 mm/min and, by reducing the traverse speed to 150 mm/min, the Warburg impedance demonstrated a slight increment. Moreover, with a further reduction in the traverse speed down to 100 mm/min, a considerable increase in Warburg impedance was observed, which is relatively comparable with the base metal. A lower Warburg value indicates a reduction in the diffusion of chloride ions through an oxide passive film and thereby improving the corrosion resistance [50,51].

It is worth noting that the constant phase element was utilized as an alternative to pure capacitance because the non-ideal capacitive behavior indicated by the deviation in phase shifts from 90°, which might be attributed to a heterogeneity property on the surface of the metal. Additionally, several models have been developed to correlate the passive film capacitance knowing the constant phase element component (admittance). Hsu and Mansfeld introduced a model (Equation (3)) to show the relationship between capacitance and admittance [52].

(3) $$C_{\mathrm{dl}}=Y_{0}\;{\left(\omega_{\max}\right)}^{n-1}$$

where, $\omega_{\max}$ is the frequency when the imaginary part of the impedance is at its max value, Y_0_ is the admittance of the CPE_dl_, and n is the value corresponding to the surface roughness.

It can be seen from Table 4 that the double-layer capacitance (C_dl_) exhibits a significant reduction for all processed samples when compared to the unprocessed sample and the values are in the order of (FP-15) > (FP-10) > (FP-25). The electrochemical behavior between the charged aluminum surface and 3.5 wt.% NaCl is reflecting an electrical dual-layer capacitance behavior [53]. Accordingly, the reduction in the double-layer capacitance of the processed surfaces indicates a corrosion resistance enhancement with increasing traverse speed, which can be attributed to a rapid decrease in surface activeness resulting from the formation of a thicker passive film [54,55]. Accordingly, the thickness of the passive film (t) can be calculated using the Helmholtz model [56] (Equation (4)).

(4) $$t\;=\frac{Ɛ\;{Ɛ}_{0}}{C_{\mathrm{dl}}\;}$$

where C_dl_ is the double-layer capacitance (µF), ${Ɛ}_{0}\;$ is the vacuum permittivity (8.85 × 10^−14^ F cm^−1^ [57]), and Ɛ is the passive layer dielectric constant (for aluminum, Ɛ=10) [57].

#### 3.3.2. Potentiodynamic Polarization (PDP)

**Figure 9:**
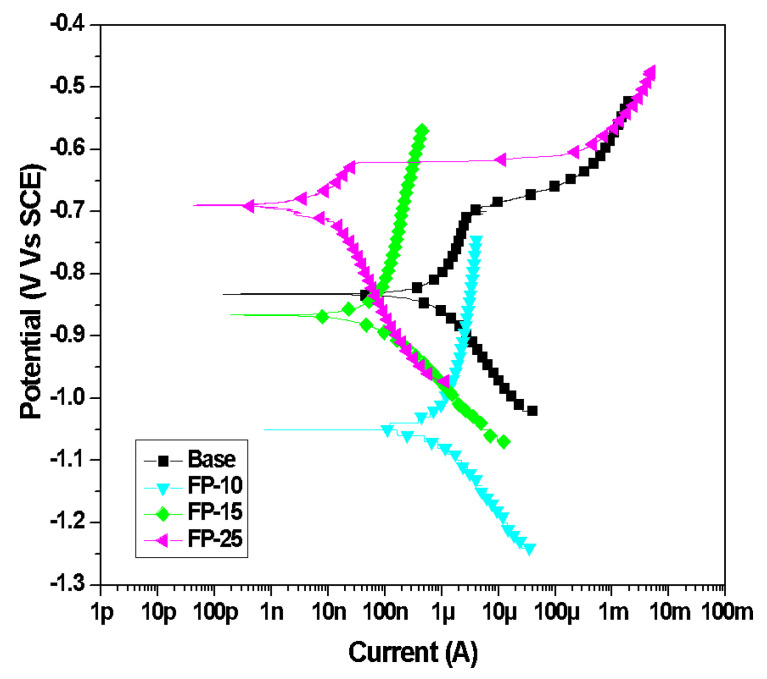
Typical PDP curves for all samples after 15 days 3.5 wt.% NaCl.

The typical PDP plots of all samples after exposure to the NaCl electrolyte are presented in Figure 9. Various electrochemical parameters, such as the anodic Tafel slope (ꞵ_a_), cathodic Tafel slope (ꞵ_c_), corrosion current density (I_corr_), and corrosion potential (E_corr_) for the base sample as well as the processed samples, were derived from the potentiodynamic polarization curves and are listed in Table 5. The polarization curves exhibited significant differences between all samples. As can be noted from the data in Table 5, there is a remarkable difference in corrosion potential between the base and processed samples. It is clear from Figure 9 and Table 5 that the corrosion potential (E_corr_) is shifted to a more positive noble potential with increasing tool traverse speed. Moreover, the FP-10 sample exhibited the lowest corrosion potential i.e the most negative, whereas the FP-25 sample showed the noblest behavior among all processed and base samples. This shift to a noble direction is an indication of an improvement in corrosion resistance. Further, as can be inferred from Figure 9 and Table 5, the corrosion current densities (I_corr_) of all processed samples were markedly lower than that of the unprocessed alloy. Additionally, a further reduction in I_corr_ can be observed as the traverse speed increases. The reduction in I_corr_. indicates that the friction stir processing for 6061 AA has enhanced its corrosion resistance. Moreover, the corrosion current density for the base sample is approximately 600 times higher than that of the FP-25 sample, which indicates the outstanding corrosion protectiveness of the FP-25 sample. Also, as compared to a base sample, the pitting potential of the FP-25 sample raised from −780 mV to −600 mV, which suggested an enhancement in the pitting resistance. Furthermore, the pitting resistance of the other two processed samples is also high despite their lower corrosion potential. Enhancing pitting resistance was also reported after the FSP of AA 7075 [58].

A remarkable variation in the cathodic and anodic Tafel slopes can be detected through the inspection of Table 5. In particular, the anodic behavior revealed that the unprocessed sample had less tendency to be oxidized compared to all processed samples, where ꞵ_a_ value for the unprocessed sample was the highest, and a further reduction in the anodic slope can be noticed with increasing tool traverse speed, which suggests an enhancement in the oxidation tendency [59]. In addition, the cathodic Tafel slope ꞵ_c_ revealed a remarkable reduction upon deploying FSP. Moreover, the cathodic slope is further reduced with increasing traverse speed, which suggests a diffusion of oxygen molecules to form (OH^−^) in the processed samples [60].

#### 3.3.3. Linear Polarization Resistance (LPR)

**Figure 10:**
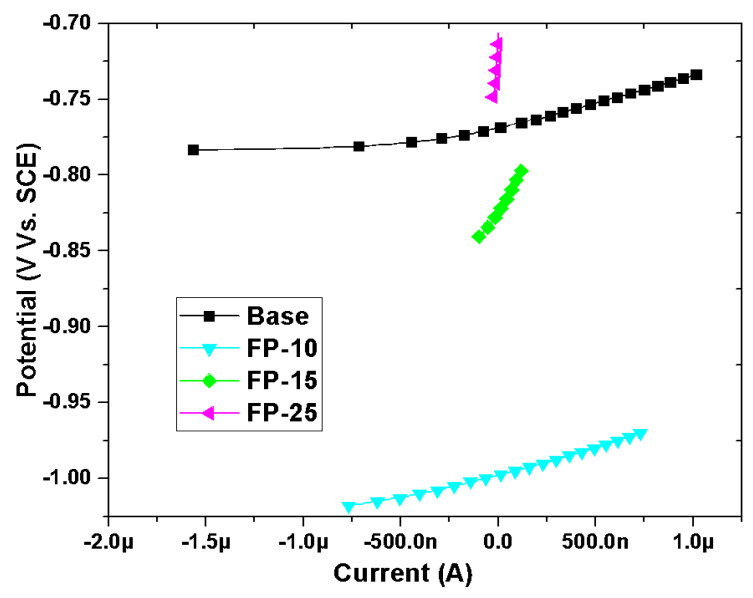
Typical LPR plots for all samples in 3.5 wt.% NaCl electrolyte.

Linear polarization plots for different samples (processed and base) in NaCl solution are presented in Figure 10. The polarization resistance (R_P_) was obtained as a slope of potential versus current. To calculate the corrosion current density based on the linear polarization resistance approach, the Stern–Geary equation was utilized, which correlates the current density (I_corr_) with polarization resistance (R_P_) (Equation (5)) [61].

(5) $$I_{\mathrm{corr}}=\frac{\beta_{a}\beta_{c}}{2.303{\;R}_{P}\left(\beta_{a}\beta_{c}\right)}$$

where R_p_ is the polarization resistance obtained from the LPR slope, and $\beta_{a}$ and $\beta_{c}$ are the cathodic and anodic Tafel slopes derived from PDP curves, respectively.

Subsequently, to calculate the corrosion rate (mpy), Equation (6) was used:

(6) $$\mathrm{CR}\;=\frac{0.131{\;I}_{\mathrm{corr}}\;\mathrm{EW}}{\rho }$$

where ρ is the sample density and EW is the sample equivalent weight.

As indicated in Table 6, the corrosion current density of the unprocessed sample is significantly higher than that of all processed samples. Additionally, the highest polarization resistance was obtained for the sample processed at the highest speed and a further reduction can be observed with a reduction in the traverse speed. It should be emphasized here that the polarization resistance is directly related to the corrosion resistance, therefore, a sample with the highest polarization resistance exhibited the highest corrosion resistance.

Results obtained from the different electrochemical techniques (LPR, EIS, and PDP) demonstrate a strong agreement. In addition, the base sample shows the highest corrosion susceptibility, while, after surface processing by employing FSP, a further increment in the corrosion resistance was observed on increasing the tool traverse speed. It should be emphasized that many studies have indicated a direct relationship between grain size, the passive film formed, and the corrosion resistance of aluminum-based alloys [62,63]. Due to the unique properties of grain boundaries compared to bulk materials, such as diffusion rates, atomic coordination, and reactivity, it is expected that a reduction in grain size will result in a substantial change in electrochemical behavior [63]. Additionally, breaking the intermetallic phases is another consequence of grain refinement [64]. These intermetallic compounds have a cathodic nature relative to the aluminum matrix and therefore their refinement and dissolution will remarkably improve the corrosion resistance. Ralston et al. [62] reported that a surface with a higher density of grain boundaries and finer grains is more likely to attract an intact passive protective film. Jilani et al. [65] have also studied the impact of grain refinement on the corrosion resistance of 1-XXX aluminum alloy. Their results confirmed that grain size reduction along with precipitate redistribution markedly improved the corrosion resistance by forming a continuous protective passive film. Likewise, the influence of severe plastic deformation of 2099 Al-Li on the formation of passive film was studied by Jinlong et al. [66]. The results illustrated that the corrosion susceptibility reduces due to refinement in both grain and precipitates, where grain refinement leads to electron work function decline and, thus, a thicker passive film.

Generally, various studies in the literature have shown that FSP resulted in an increase in the fraction of high-angle grain boundaries [67,68,69]. For instance, Li et al. [69] studied the impact of heat input during FSP on Mg-Li alloy on the fraction of high-angle grain boundaries. Their results indicated that the reduction in grain size and the increase in the fraction of high-angle grain boundaries are associated with lower heat input.

The consequence of this shift to a higher fraction of high-angle grain boundaries (HAGBs) on corrosion behavior was intensively investigated by Argade et al. [70], Dan et al. [71], and Rao et al. [60]. For example, Argade et al. [70] have concluded that the corrosion enhancement after friction stir processing of AA 5083 was attributed to grain refinement, which improved the polarization resistance, passivation, and pitting potential. Moreover, high-angle grain boundaries provided by the process raise the corrosion resistance by accelerating the passivation re-passivation phenomenon. Dan et al. [5] have studied the impact of grain refinement on the corrosion behavior of pure aluminum and have reported a significant enhancement in corrosion and pitting resistance for the sample with finer grain, which was attributed to a denser passive film. Further, it was also suggested that the oxide film favored a higher grain boundary, which protects the surface of the processed sample against chloride attack, thereby enhancing the corrosion resistance. Rao et al. [60] have demonstrated that the transformation from low-angle grain boundaries to high-angle counterparts after friction stir processing of Al–30Si alloy is a fundamental reason for the stability of the oxide film formed. Therefore, the results obtained in the current study may follow the same behavior, whereby employing FSP significantly diminishes grain size and raises the fraction of high-angle grain boundaries. Moreover, a further reduction in grain size was detected with increasing tool traveling speed, which resulted in superior corrosion resistance due to the more adhered and compacted passivation film.

### 3.4. SEM Images of the Corroded Samples

**Figure 11:**
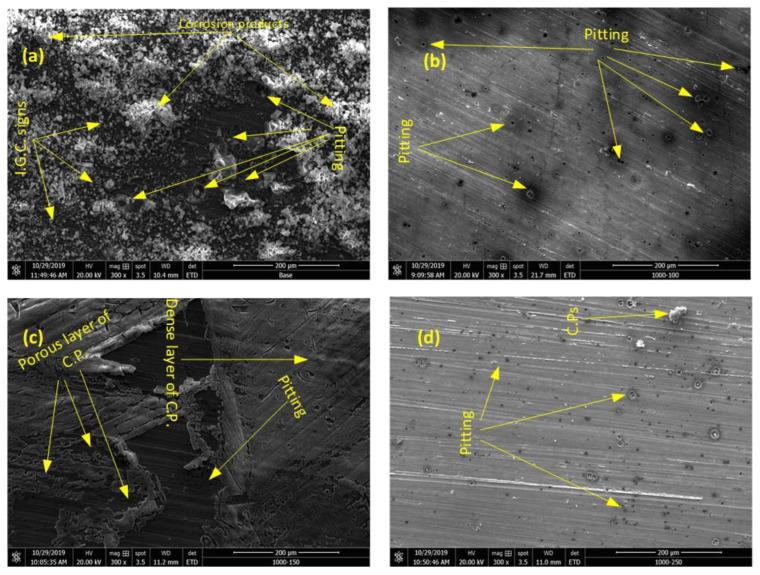
SEM images of the corroded surface of (**a**) base sample, (**b**) FP-10 sample, (**c**) FP-15 sample, and (**d**) FP-25 sample in 3.5 wt.% NaCl.

The morphology of the intense corrosion attack of the processed and base samples after 15 days of exposure to the electrolyte is depicted in Figure 11. For the base sample, a severe corrosion attack on the surface can be clearly noticed and indicated by corrosion products adhered to the surface. Additionally, within the corrosion products, large pits are observed. Surprisingly, microcracks can also be detected beneath the corrosion products, which may be ascribed to the initiation of intergranular corrosion. The corrosion marks of the friction-stir-processed samples demonstrated a different appearance, as shown in Figure 11b–d. The size and the density of pits became noticeably lower compared with that of the base sample. Moreover, the adsorption of corrosion products is hardly detected on samples processed at 100 and 250 mm/min, as shown in Figure 11b,d, which is attributed to a grain refinement that enhanced the passivation film formed. Interestingly, the adhesion of corrosion products can be seen on the surface of the FP-15 sample in Figure 11c; however, one with completely distinctive characteristics compared to the base counterpart which can be interpreted by the reduction in the outer film resistance of this sample (90 Ω). In addition, a dense layer of corrosion products may act as a barrier to impede a further corrosion attack.

## 4. Conclusions

Friction stir processing using a pinless tool was successfully applied under different tool traverse speeds on structural aluminum alloy 6061. The results have shown a remarkable influence of FSP on the modified surface microstructure, mechanical properties, and corrosion resistance. From the current study, the following conclusions can be drawn:

1. It was found that friction stir processing resulted in considerable grain refinement of the base material of up to 81, 82.5, and 83.4 % reduction in the grain size at a traverse speed of 100, 150, and 250 mm/min, respectively. In addition, increasing the traverse speed from 100 to 250 mm/min has only a slight influence on the grain size refinement. Consequently, the microhardness was generally enhanced compared to the base material. However, increasing the traverse speed between 150 and 250 mm/min resulted in a reduction in the microhardness properties. This was attributed to a lower heat input at higher traverse speeds.
2. All the employed electrochemical techniques used in testing the corrosion resistance of all samples proved the achieving of an outstanding corrosion resistance behavior after deploying FSP on the 6061-aluminum base substrate, and an increase of the traverse speed caused a rapid improvement in the corrosion resistance.
3. Estimation of the passivation film thickness from the EIS measurements showed that the film thickness increased from 2.7 nm of the base materials to 7.6, 8.9, and 12.2 nm for traverse speeds of 100, 150, and 250 mm/min, respectively. This led to an improvement in the corrosion behavior of the samples by reducing the corrosion rate by 47.9, 96.6, and 99.8% for traverse speeds of 100, 150, and 250 mm/min, respectively, according to the potentiodynamic measurement.

## Figures and Tables

**Table 1:** Chemical composition of 6061 AA.

Element	Mg	Si	Cu	Fe	Mn	Al
wt.%	0.82	0.71	0.23	0.63	0.14	Bal.

**Table 2:** Process conditions and sample designations.

Samples	Rotation Speed (rpm)	Traverse Speed (mm/min)
FP10	1000	100
FP15		150
FP25		250

**Table 3:** Electrochemical parameters obtained from simulating the experimental EIS data.

Samples	Rs (Ω)	Y_n_ (10^−6^) (Ss^a^)	n	R_f_ (kΩ)	Y_c_ (10^−6^) (Ss^a^)	c	R_ct_ (kΩ)	W (10^−5^) Ss^1/2^	χ (10^−4^)
Base	12.38	23.5	0.924	-			6.14	41.4	29.10
FP-10	11.26	10.94	0.878	1.59	3.92	0.98	18.7	56.7	4.68
FP-15	12.24	6.94	0.921	0.09	5.25	0.93	83.9	7.26	15.96
FP-25	12.79	4.43	0.892	1.72	2.16	0.97	1500	6.80	15.29

**Table 4:** Variation in double-layer capacitance and passive film thickness for different samples.

Samples	C_dl_ (µF)	t (nm)
Base	33.3	2.65
FP-10	11.6	7.63
FP-15	9.99	8.86
FP-25	7.28	12.16

**Table 5:** Electrochemical parameters obtained from PDP plot.

Samples	β_a_	β_c_	I_corr_ (µA)	E_corr_ (V)	Corrosion Rate (mpy)
Base	1.994	2.78 × 10^−1^	3.28	−0.832	1.498
FP-10	7.38 × 10^−1^	1.68 × 10^−1^	1.7	−1.05	7.79 × 10^−1^
FP-15	6.57 × 10^−1^	1.10 × 10^−1^	1.12 × 10^−1^	−0.867	5.11 × 10^−2^
FP-25	8.69 × 10^−2^	7.60 × 10^−2^	5.47 × 10^−3^	−0.693	2.50 × 10^−3^

**Table 6:** LPR data obtained after 15 days of exposure of all samples to 3.5 wt.% NaCl solution.

Sample	R_p_ (kΩ)	I_corr_ (μA)	CR (mpy)
Base	35.87	2.724	1.168
FP-10	34.63	1.72	0.736
FP-15	220.1	0.185	0.0795
FP-25	2087	0.00844	0.00362

## Data Availability

The raw/processed data required to reproduce these findings cannot be shared at this time as the data also forms part of an ongoing study.

## Supplementary Materials

The following supporting information can be downloaded at: https://www.mdpi.com/article/10.3390/ma15228124/s1, Figure S1: Optical micrograph for FP-10 sample to demonstrate the method adopted to measure the mean grain size; Figure S2: Optical micrograph for FP-15 sample to demonstrate the method adopted to measure the mean grain size; Figure S3: Optical micrograph for FP-25 sample to demonstrate the method adopted to measure the mean grain size; Figure S4: Optical micrograph for base sample to demonstrate the method adopted to measure the mean grain size; Table S1: Optical micrograph for FP-10 sample to demonstrate the method adopted to measure the mean grain size; Table S2: Typical grain morphology information for FP-15 sample drawn from the Olympus stream analysis software; Table S3: Typical grain morphology information for FP-25 sample drawn from the Olympus stream analysis software; Table S4: Typical grain morphology information for the base sample drawn from the Olympus stream analysis software.
