# Supplementary material for: Influence of Friction Stir Surface Processing on the Corrosion Resistance of Al 6061
Source: Materials (Basel). 2022 Nov 16;15(22):8124. doi: 10.3390/ma15228124 (PMC9696715; doi:10.3390/ma15228124)
Supplement: Supplementary file 1 [file materials-15-08124-s001.zip › materials-1956040-supplementary.pdf]

Table S1: Typical grain morphology information for FP-10 sample drawn from the Olympus stream analysis software

Reference	FP-10	
Standard	ASTM E 112-13	
Grain Size Number G	8.35	
Mean Intercept Length [µm]	17.74	
Elongation	0.98	
Average Number of Intercepts	180.00	
Number of Intercepts per Unit Length [1/mm]	56.36	

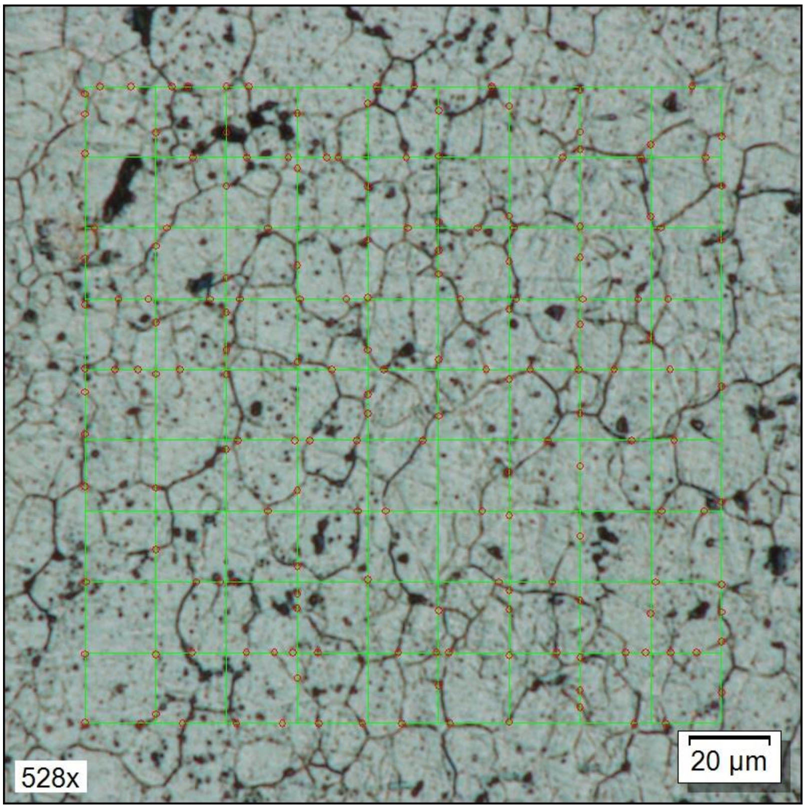

Figure S1: Optical micrograph for FP-10 sample to demonstrate the method adopted to measure the mean grain size

Table S2: Typical grain morphology information for FP-15 sample drawn from the Olympus stream analysis software

Reference	FP-15
Standard	ASTM E 112-13
Grain Size Number G	8.58
Mean Intercept Length [µm]	16.38
Elongation	0.91
Average Number of Intercepts	195.00
Number of Intercepts per Unit Length [1/mm]	61.06

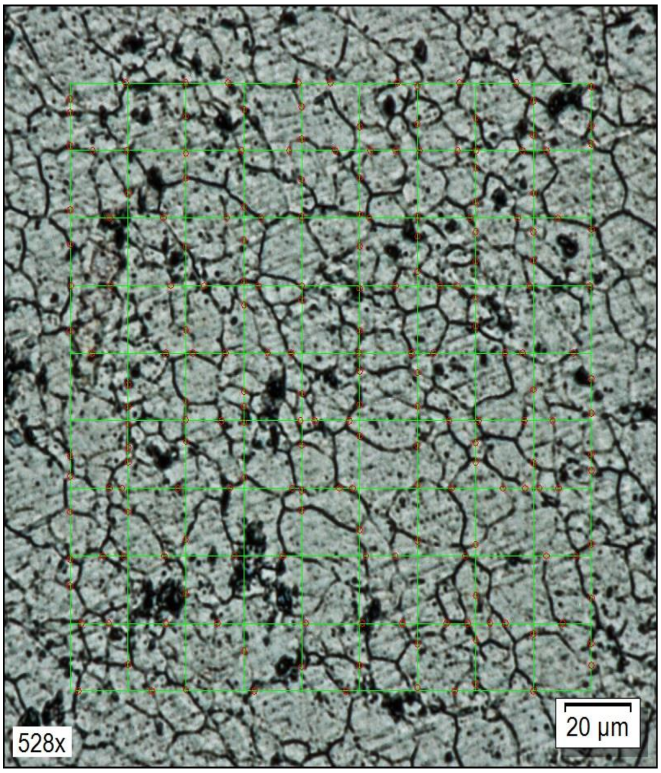

Figure S2: Optical micrograph for FP-15 sample to demonstrate the method adopted to measure the mean grain size

Table S3: Typical grain morphology information for FP-25 sample drawn from the Olympus stream analysis software

Reference	FP-25
Standard	ASTM E 112-13
Grain Size Number G	8.72
Mean Intercept Length [µm]	15.58
Elongation	1.09
Average Number of Intercepts	205.00
Number of Intercepts per Unit Length [1/mm]	64.19

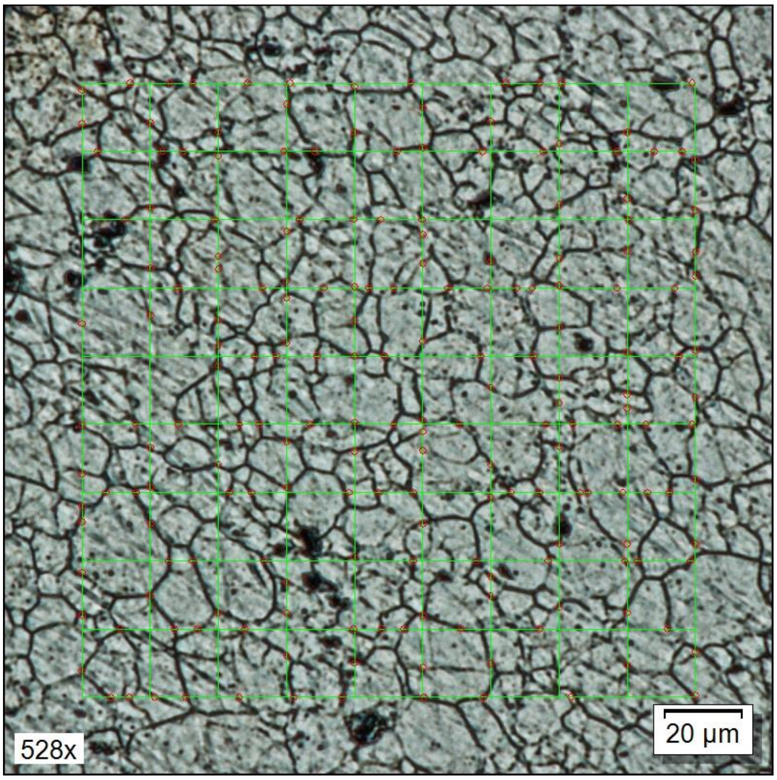

Figure S3: Optical micrograph for FP-25 sample to demonstrate the method adopted to measure the mean grain size

Table S4: Typical grain morphology information for the base sample drawn from the Olympus stream analysis software

Reference	BASE
Standard	ASTM E 112-13
Grain Size Number G	3.54
Mean Intercept Length [µm]	93.93
Elongation	0.74
Average Number of Intercepts	68.00
Number of Intercepts per Unit Length [1/mm]	10.65

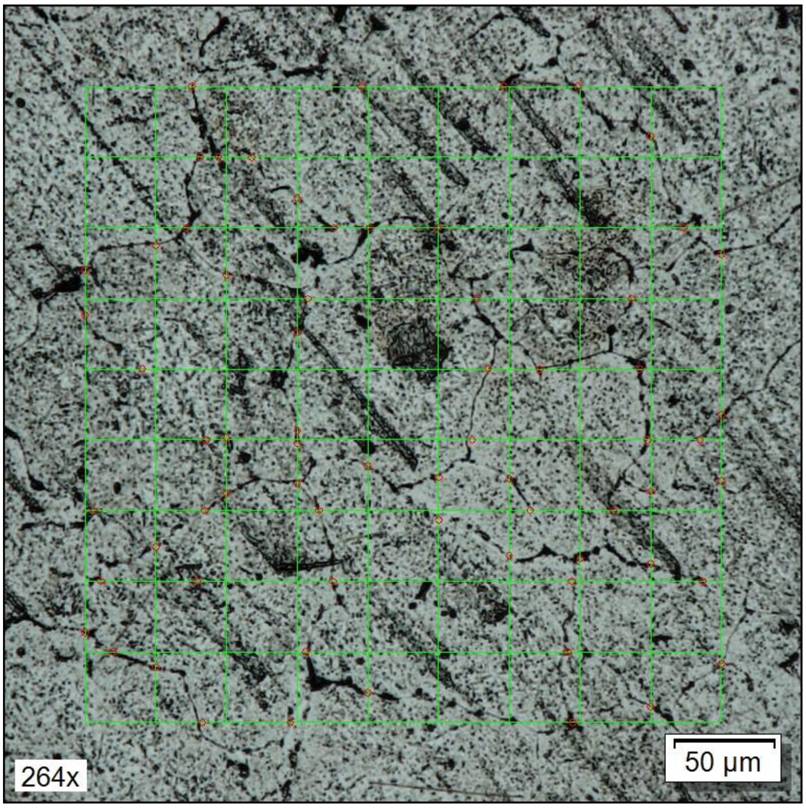

Figure S4: Optical micrograph for base sample to demonstrate the method adopted to measure the mean grain size
